# Effect of the Type and Number of Adverse Childhood Experiences and the Timing of Adverse Experiences on Clinical Outcomes in Individuals with Bipolar Disorder

**DOI:** 10.3390/brainsci10050254

**Published:** 2020-04-27

**Authors:** Young-Min Park, Tatyana Shekhtman, John R. Kelsoe

**Affiliations:** 1 Department of Psychiatry, Ilsan Paik Hospital, Inje University College of Medicine, Goyang 10380, Korea; 2 Deparrtment of Psychiatry, University of California San Diego, San Diego, CA 92093, USA; tashek13@gmail.com

**Keywords:** bipolar disorder, adverse childhood experiences, abuse, childhood life events scale, clinical outcome/prognosis

## Abstract

Studies have reported an association between adverse childhood experiences (ACEs) and the clinical outcomes of bipolar disorder (BD). However, these studies have several limitations; therefore, we aimed to clarify the effect of the type and number of ACEs and the timing of adverse experiences on clinical outcomes in patients with BD. We analyzed the data of patients with BD (*N* = 2675) obtained from the National Institute of Mental Health: Bipolar Disorder Genetic Association Information Network, Translational Genomic Institute-I, and Translational Genomic Institute-II. All patients had been diagnosed using the Diagnostic Interview for Genetic Studies. ACEs were evaluated using the Childhood Life Events Scale (CLES). We analyzed the relationship between childhood trauma and clinical outcome in patients with and without exposure to ACEs. We found that ACEs had a robust negative effect on clinical outcomes, including earlier age at onset, presence of psychotic episodes, suicide attempts, mixed symptoms or episodes, substance misuse comorbidity, and worse life functioning. Specifically, the number of ACEs had the most significant effect on clinical outcomes; however, specific ACEs, such as physical abuse, had a considerable influence. Moreover, post-childhood adverse experiences had a weaker effect on clinical outcomes than ACEs did. There was an association of ACEs with negative clinical outcomes in patients with BD. This indicates the importance of basic and clinical research on ACEs in patients with BD.

## 1. Introduction 

Adverse childhood experiences (ACEs) are potentially traumatic events that can have negative, lasting effects on health and well-being [1]. ACEs range from physical, emotional, or sexual abuse and neglect to the incarceration of a parent or parental divorce [1]. There is evidence indicating ACEs as a risk for the development of BD and of worse outcomes for bipolar disorder (BD) [2]. A study conducted two decades ago reported a higher number of ACEs in patients with BD than in normal controls [3]. Furthermore, adverse experiences, including childhood abuse and neglect, have been reported in more than half of the patients with BD [4]. Subsequent studies have also indicated a relationship between ACEs and BD [2]. Additionally, there is a reported association between ACEs and the clinical outcomes of BD, including early age at onset (AAO), psychotic features, rapid cycling, a higher mood episode number, and suicidality [4,5,6,7].

However, the validity of these findings is uncertain considering the limitations of the aforementioned studies [8]. For example, more than 40% of the studies did not use structured clinical interviews for BD diagnosis; furthermore, they did not use scales for ACEs evaluation, including the Childhood Trauma Questionnaires (CTQ) and Childhood Life Events Scale (CLES) [3,9]. These aforementioned limitations contribute to the inconsistent results and their reduced reliability. Furthermore, there are scarce large-scale studies on the ACE types affecting the clinical outcomes, the effect of post-childhood abuse or adverse experiences on clinical outcomes, and the influence of the number of ACEs on clinical outcomes. We hypothesized that specific ACEs type or multiple ACEs interact with the vulnerability to BD, and that this interaction would induce earlier AAO and worse clinical outcomes of BD. We also hypothesized that post-childhood adverse experiences had a weaker effect on clinical outcomes than ACEs did.

The aim of this study was to clarify the relationship between ACEs and the clinical outcomes of BD. Thus, we investigated the effects of various ACEs, including the number, type, and timing, on the clinical outcomes of BD.

## 2. Methods

### 2.1. Participants

We obtained the clinical data of 2675 bipolar patients with ACEs data from the Bipolar Genomic Study (BiGS) [10] by Bipolar Disorder Genetic Association Information Network and Translational Genomic Institute which is a large sample that was collected by the National Institute of Mental Health Genetics Initiative for Bipolar Disorder for large-scale genome-wide association study (GWAS) in five waves at 11 sites across the United States. All subjects provided written informed consent in accordance to protocols from local institutional review boards. The subjects’ data were obtained by Dr. Kelsoe, who is a member of the BiGS.

### 2.2. Diagnostic Interview for Genetic Studies and Childhood Life Events Scale 

All patients were evaluated using the Diagnostic Interview for Genetic Studies (DIGS) [11,12], which is a specific clinical interview for assessing major mood and psychotic disorders, as well as their spectrum conditions through a semi-structured design corresponding to a wide spectrum of DSM-IV criteria [11]. Information was obtained from medical records and family informants and it was reviewed along with the interview by a panel of experienced clinicians to obtain a final best-estimate diagnosis. We used the following information from the DIGS data: AAO, chronicity presence; psychotic symptom; history of mixed symptoms or episodes; suicidality assessment; psychosis history; general impact of illness on life functioning; and substance misuse history (Table 1). We evaluated ACEs using CLES [9], which is a nine-item scale that assesses ACEs occurring between the age of 3 and 12 years. The CLES is scored as the accumulated number of ACEs during that period (range: 0–9 events). The CLES contains the following items: death of a parent, death of a sister or brother, onset of a chronic illness (e.g., juvenile diabetes), long-term hospitalization (≥1 month), permanent injury or disability (e.g., loss of a limb), physical abuse (PA), receiving a death or injury threat, leaving home unexpectedly (e.g., foreclosure by the bank), and serious unexpected life changes (e.g., a parent losing a job).

### 2.3. Statistical Analyses 

We divided the patients into two groups based on the exposure to ACEs (no ACEs vs. ACEs or no PA vs. PA), into three groups based on the timing of adverse experiences (no experiences vs. post-childhood (after 12 years) experiences or adverse experiences vs. ACE), and into four groups based on the number of different ACE types (none vs. one vs. two vs. ≥ three types) using the CLES score. Except for AAO, all the other continuous variables were non-normally distributed. We analyzed the continuous variables using the Mann–Whitney and Kruskal–Wallis tests, as well as the t-test and ANOVA. We used the chi-squared test to analyze categorical variables. Moreover, we conducted multivariate regression analyses and logistic regression to investigate the association of the number of ACEs, specific ACEs, and the timing of adverse experiences with AAO and clinical outcome severity. We conducted all statistical analyses using SPSS (version 21; IBM, Armonk, NY, USA) and SALT (version 2.5; Istech Inc., Goyang, Republic of Korea); we considered *p* < 0.05 as statistically significant.

## 3. Results

Table 2 presents the demographic and clinical characteristics of all patients. The mean age and AAO of the patients were 44.1 years and 18.5 years, respectively. More than half of the patients had a history of psychotic episodes, suicide attempts, or comorbidity of substance misuse. Furthermore, 49.6% of the patients had a history of mixed symptoms or episodes. In addition, 63.1% of the patients had experienced at least one ACE; moreover, 39.3% of them reported having a PA experience.

### 3.1. Relationship between the Number of Adverse Childhood Experiences and the Clinical Outcomes of Bipolar Disorder

We found an association of ACE presence or absence or the ACE frequency with the clinical outcomes of BD (Table 3 and Table 4). Patients with ACEs had a significantly earlier AAO than those without ACEs (Table 3). Furthermore, there was a significant relationship between the number of ACEs and AAO (Table 4) and between the number of ACEs and annual manic or depressive episodes (Table 4). There was a significantly higher proportion of female patients in the group with ACEs than in that without ACEs (Table 3 and Table 4). The significant difference remained after adjustment for age and sex (Table 4). Notably, exposure to at least two ACEs contributed to the presence of psychotic episodes in the majority of the patients (Table 3). We obtained similar results after assessing these clinical manifestations using DIGS (continuous variables).

We used logistic regression analyses to assess the number of ACEs based on the presence or absence of psychotic features, suicide attempts, mixed symptoms or episodes, comorbidity of substance misuse, and worsening of life functioning. We found that there were significant relationships between the number of ACEs and the aforementioned clinical manifestations (Table 5). Specifically, we found an odds ratio of 329 for patients with two ACEs and a history of psychotic episodes compared to patients with no ACEs (Table 5).

### 3.2. Effect of the Type of Adverse Childhood Experiences and the Timing of Adverse Experiences on the Clinical Outcomes of Bipolar Disorder

We divided the patients into three groups (no adverse experience, post-childhood adverse experience, and ACEs groups) based on the nine ACE types in CLES. There were significant group differences in the AAO and DIGS scores regarding the chronicity, psychosis, mixed symptoms, suicidality, substance misuse, and worsening of life functioning (Table 6). Multiple comparisons indicated that most clinical outcomes in the ACEs group were worse than those in the group without ACEs; furthermore, there was a significant among-group difference. Moreover, some clinical outcomes were worse in the group with adverse post-childhood experience than those in the group without ACEs; however, there was no difference in the remaining clinical outcomes.

PA is the most prevalent item among nine ACEs (Table 6). PA and death or injury threats were the most potent factors among all ACE types considering that they affected all clinical outcomes of DIGS (Table 6). Table 7 shows the odds ratios for the groups with childhood or post-childhood PA and the clinical outcomes (categorical variables) of BD compared with those for the group with no PA. The group with childhood PA had significant odd ratios for all clinical outcomes; furthermore, it had the highest odds ratio for psychotic features (OR = 5.19 [3.89–6.92]). In addition, the group with post-childhood PA had a significant odds ratio for suicidality (OR = 2.50 [1.12–5.59]) (Table 6).

### 3.3. Effect of Physical Abuse and the Number of Adverse Childhood Experiences on the Clinical Outcomes of Bipolar Disorder

**Figure 1:**
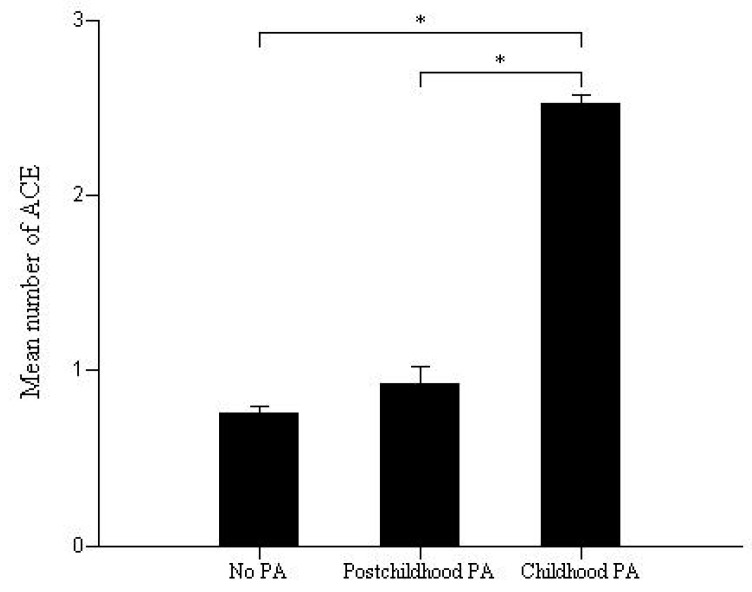
Comparisons of the number of adverse childhood experiences among the groups with childhood physical abuse, with post-childhood physical abuse, and without physical abuse (* *p* < 0.01).

Compared to other ACEs, PA was the most potent factor affecting clinical outcomes (Table 6). The number of ACEs was higher in the group with childhood PA than that in the groups with post-childhood PA and without childhood PA (Figure 1).

In the current study, both of the number of ACEs and PA were significantly associated with clinical outcomes. Thus, we conducted multiple linear regression to analyze the effect of the number of ACEs and the presence of childhood PA to determine which of them affects clinical outcomes more (Table 8). We found a positive correlation between the number of ACEs and all clinical outcomes except for mixed symptoms. Moreover, the presence of childhood PA affected AAO and worsened the severity of mixed symptoms, suicidality, and substance misuse. Finally, AAO had an effect on chronicity, psychosis, mixed symptoms, suicidality, and life functioning.

## 4. Discussion

We aimed to clarify the relationship between ACEs and the clinical outcomes of BD. Moreover, we analyzed the effects of various ACE factors, including the number, type, and timing, on the clinical outcomes of BD.

We found that 63.1% of the patients had a history of exposure to at least one ACE. A previous review reported that 67% of patients with BD across 16 studies had a history of exposure to at least one ACE [13], which is consistent with our findings; however, the prevalence ranged from 5.3% to 76.5%. This significant discrepancy could be attributed to methodological limitations in some of the studies, including small sample sizes, as well as a lack of the use of scales for ACE evaluation and of structured clinical interviews for BD diagnosis [8]. In addition, the female group reported greater frequencies of ACEs than the male group. Moreover, the female group with ACEs revealed stronger associations with suicidality than the male group.

Moreover, we observed that several ACE types, including family loss, chronic illness, lengthy hospitalization, permanent injury or disability, adverse life changes, and PA, have robust negative effect on clinical outcomes, including AAO, psychotic episodes, chronicity, suicide attempts, mixed symptoms or episodes, comorbidity of substance misuse, and life functioning.

Furthermore, we observed a significant positive correlation between the number of ACEs and the severity of clinical manifestations (Table 4 and Table 5). A previous study reported a more significant effect of abuse than that of neglect [6]. Contrastingly, we found a positive correlation between the number of ACEs and the severity of the clinical outcomes of BD, which is consistent with a previous study (Table 4 and Table 5) [4]. This indicates that the number and severity of ACEs influence the clinical outcomes of BD. Our results are consistent with those of previous reports of serious clinical outcomes in patients with BD exposed to ACEs; however, the variables with a significant effect on the clinical features, course, and comorbidities have varied across studies [4,6,14,15,16,17,18,19,20].

We found an association between exposure to ACEs and early AAO. Specifically, there was a positive correlation of the number of ACEs and specific ACEs, including PA, with early AAO. This suggests that persistent hypothalamic-pituitary-adrenal axis over-activation leads to an elevated risk for BD during adolescence or adulthood [21]. Another suggestion could be that ACEs affects the BD onset by interrupting brain network maturation [22], impairing the limbic-cortical circuits (e.g., the volume reduction of the hippocampus and amygdala) [23,24], and interacting with genetic or epigenetic factors [25].

Regarding psychotic features, 69.4% of our patients had experienced at least one psychotic episode during their lifetime. Interestingly, there was a high odds ratio for patients with more than one ACE to have a history of psychotic episodes compared to patients without a history of ACEs (Table 5). Furthermore, 99.8% of patients with an experience of more than two ACEs had at least one psychotic episode; contrastingly, the concomitant prevalence in patients with no ACE history was approximately 50% (Table 4). Therefore, psychotic symptoms were more easily affected by the presence and number of ACEs compared with other clinical outcomes. Psychotic symptoms could be associated with dopaminergic abnormalities induced by HPA axis over-activation [13]. Previous studies have also reported a significant association of psychotic features in patients with BD with exposure to ACEs [15,26]. Contrastingly, other previous studies reported that exposure to ACEs only elevated psychotic symptom occurrence regardless of the psychiatric diagnosis [5,14,20]. Therefore, it remains unclear whether ACEs influences psychotic episode occurrence or psychotic symptoms regardless of the psychiatric diagnosis in patients with BD.

Similarly, we observed a positive correlation between the number of suicide attempts and the number of ACEs. In addition to exposure to ACEs, being female was associated with an increased number of suicide attempts. This could be attributed to the number of ACEs being higher among females. However, a meta-analysis on patients with BD by Schaffer et al. reported an association of being female with suicide attempts and of being male with suicide completion [27]. Furthermore, being younger had an effect on suicide attempts; however, the odds ratio was only 0.99. However, after adjusting for sex and age, the association of ACEs with suicide attempts remained. Therefore, ACEs, as well as being female and younger, could have an additive effect on suicide attempts in patients with BD, which is similar to the findings of the report by Etain et al [6].

Additionally, there was an association of mixed symptoms or episodes with ACEs. However, after adjustment for age, sex, AAO, and PA, unlike for other clinical outcomes, there was no relationship between the number of ACEs and mixed symptoms (Table 8). However, the presence of PA and earlier AAO had an effect on the presence of mixed symptoms/episodes. Therefore, considering the constant odds ratio despite the increased number of ACEs, it is possible that mixed symptoms/episodes are affected by specific ACEs rather than the number of ACEs (Table 5 and Table 6).

Moreover, there was a positive association between substance misuse and ACEs, which remained even after adjustment for sex and age. In addition to ACEs, being male was associated with an increased frequency of substance abuse or dependence. Therefore, ACEs, as well as being male, had an effect on substance misuse in patients with BD. Furthermore, there was a positive correlation between the number of ACEs and substance misuse.

There was a significant relationship between ACEs and life functioning; furthermore, patients with more than two ACEs had worse life functioning. In addition to ACEs, being male was associated with worse life functioning. This suggests that ACEs could contribute to worse social and cognitive function, which is consistent with previous findings [19].

Various types of ACEs, including PA, had an effect on clinical outcomes in patients with BD. However, PA (item 6) and death or injury threat (item 7) were the most potent factors among all types of ACEs since they affected all clinical outcomes (Table 6). However, multiple regression analysis revealed that the number of ACEs, rather than a specific ACE, such as PA, could be a more potent factor influencing clinical outcomes in patients with BD. However, PA does not represent a single ACE; rather, it represents a complex collective of ACEs since the group with childhood PA had more ACEs than the groups with post-childhood PA or without PA did (Figure 1). Therefore, future studies should assess whether a specific ACE or the number of ACEs had the most significant effect on clinical outcomes.

This study has several limitations. First, we retrospectively obtained data regarding ACEs using CLES. Studies have used varying ACEs evaluation techniques, such as chart reviews, clinical interviews, CTQ, and CLES, which has resulted in heterogeneity in the definition and measurement of childhood trauma [8]. For example, CLES focuses on a diverse range of ACEs, such as the loss of parents and siblings, illness, hospitalization, permanent injuries, and PA. Contrastingly, the CTQ reflects a more specific trauma type with the main focus on abuse or neglect. However, despite the methodological differences, a majority of previous studies reported similar results, including earlier AAO and more severe clinical outcomes of BD in patients exposed to ACEs, with recent studies (including the present one) reporting consistent findings. Second, CLES has not been validated to date. In addition, nine adversities are measured and the sum of the number of adversities was presented as a score. Third, the current study included a much larger female sample than male sample. However, gender was included as a covariate in the analyses. Future studies should investigate the frequency and vulnerability to ACEs according to gender differences. Forth, the post-childhood adversities have occurred after the onset of bipolar disorder in some subjects. Lastly, this study was designed without considering a selection bias. Thus, future studies need to be performed with careful consideration of selection bias. Despite these limitations, our study has several strengths, including a large sample size and thorough assessments of the data of patients with BD obtained using structured questionnaires.

In conclusion, we found an association of ACEs with a robust negative effect on clinical outcomes, including AAO, psychotic episodes, suicide attempts, mixed symptoms or episodes, comorbidity of substance misuse, and worse life functioning. These findings indicate the clinical importance for studies on BD to evaluate ACEs. Future prospective case-control studies should attempt to confirm these relationships.

## Figures and Tables

**Table 1:** Scoring of diagnostic interview for genetic studies according to clinical outcomes.

Score	Chronicity	Psychotic Symptoms	Mixed Symptoms	Suicidality	General Impact of Illness on Life Functioning	Substance Misuse
0	never	never	never	never	never	never
1	duration < 2 years	fleeting	mixed symptoms	passive death wishes	employment	no dependence
2	duration ≥ 2 years	one episode	≥ a mixed episode	thought about suicide	employment but not disabled	brief usage
3	frequent symptoms	≥2 episodes		acted on ambivalently	disabled but living independently	relapsing
4		all episodes		acted on seriously	disabled & not living independently	chronic usage
5		chronic psychosis				

**Table 2:** Demographic and clinical characteristics of patients with bipolar disorder (BD) (*N* = 2675).

Variable	Value
Age, years (mean ± SD) (age range)	44.1 ± 13.0 (17–83)
Sex ratio, males/females (%)	36.3/63.7
Race (%)	
White	80.9
Other	19.1
Illness subtype (%)	
Bipolar I	94.9
Bipolar II	3.4
Bipolar NOS	1.7
Mean number of ACEs (CLES score) (mean ± SD)	1.4 ± 1.5
AAO, years (mean ± SD)	18.5 ± 9.4
Presence of psychotic episodes (%)	69.4
History of suicide attempts (%)	50.9
Presence of substance misuse (%)	59.3
Presence of mixed symptoms or episodes (%)	49.6
Mean number of manic episodes (per year) (mean ± SD)	0.5 ± 1.2
Mean number of depressive episodes (per year) (mean ± SD)	0.9 ± 2.2
History of physical abuse (%)	39.3
Frequency of the number of ACEs (%)	
0	36.9
1	23.8
2	17.8
≥3	21.5

Data are mean ± SD or percentage values. SD, standard deviation; CLES, Childhood Life Events Scale; ACEs, adverse childhood experiences; NOS, not otherwise specified; AAO, age at onset.

**Table 3:** Comparison of demographic and clinical variables between groups with and without adverse childhood experience, defined according to CLES scores.

Variable	Group without ACE	Group with ACE	*P*
AAO, years (mean ± SD)	20.1 ± 9.7	17.5 ± 9.0	<0.01^a^
Age at assessment, years (mean ± SD)	43.7 ± 14.1	44.3 ± 12.1	0.28^b^
Sex ratio, males/females (%)	42.5/57.5	32.3/67.7	<0.01^c^
Number of manic episodes^*^ (mean ± SD)	0.5 ± 0.9	0.6 ± 1.4	0.30^b^
Number of depressive episodes^*^ (mean ± SD)	1.0 ± 2.8	0.9 ± 1.7	0.21^b^
History of psychotic episodes (mean ± SD)	47.5	82.4	<0.01^c^
History of suicide attempts (%)	75.7	82.4	<0.01^c^
History of mixed symptoms or episodes (%)	44.9	52.4	<0.01^c^
Presence of substance misuse (%)	54.3	62.2	<0.01^c^
Presence of worsening in life functioning (%)	81.7	86.7	<0.01^c^

Data are mean ± SD or percentage values, ^*^ per year. AAO, age at onset; ACEs, adverse childhood experiences. ^a^ Independent t-test, ^b^ Mann-Whitney test, ^c^ goodness-of-fit test.

**Table 4:** Comparison of demographic and clinical variables between groups with no adverse childhood experiences and one, two, and three or more types of adverse childhood experiences, defined according to CLES.

Variable	No ACE (*n* = 987)	One Type of ACE (*n* = 637)	Two Types of ACE (*n* = 477)	Three and More Types of ACE (*n* = 574)	*P*	*P* ^d^
AAO, years (mean ± SD)	20.1 ± 9.7	18.9 ± 9.1	17.1 ± 9.1	16.3 ± 8.7	<0.01^a^	<0.01
Age at assessment, years (mean ± SD)	43.7 ± 14.1	43.0 ± 12.9	45.0 ± 11.7	45.3 ± 11.3	0.057^b^	NA
Sex ratio, males/females (%)	42.5/57.5	31.0/69.0	32.3/67.7	34.0/66.0	<0.01^c^	NA
Mean episodes per year (mean ± SD)	1.5 ± 3.0	1.3 ± 2.3	1.5 ± 3.1	1.5 ± 1.9	<0.05^c^	<0.05
History of psychotic episodes (%)	47.5	55.2	98.6	99.8	<0.01^c^	<0.01
History of suicide attempts (%)	75.7	79.8	84.1	83.9	<0.01^c^	<0.01
History of mixed symptoms (%)	44.9	51.8	51.5	53.8	<0.01^c^	<0.01
Presence of substance misuse (%)	54.3	57.2	60.3	69.6	<0.01^c^	<0.01
Presence of worsening in life functioning (%)	81.7	84.0	85.3	91.4	<0.01^c^	<0.01

Data are mean ± SD or percentage values. AAO, age at onset; NA, not applicable; ACEs, adverse childhood experiences. ^a^ ANOVA, ^b^ Kruskal-Wallis test, ^c^ Fisher’s exact test, ^d^ Statistical significance after controlling for age and sex.

**Table 5:** Individual odds ratios for the number of adverse childhood experiences and the clinical outcomes of BD compared to the group with no adverse childhood experiences (reference).

Variable (Categorical Variables)	Coefficient	SE	Wald	*P*	OR (95% CI)
^a^ Psychotic episodes					
One type of ACE	0.35	0.14	6.65	<0.05	1.42 (1.09–1.86)
Two types of ACE	5.80	1.01	33.22	<0.01	329.24 (45.86–2363.70)
Three or more types of ACE	12.62	29.20	0.00	0.67	NA
^a^ Mixed symptoms					
One type of ACE	0.46	0.14	10.65	<0.01	1.59 (1.20–2.09)
Two types of ACE	0.45	0.16	8.10	<0.01	1.52 (1.15–2.12)
Three or more types of ACE	0.42	0.15	7.68	<0.01	1.52 (1.13–2.06)
^a^ Suicide attempts					
One type of ACE	0.12	0.16	0.50	0.48	1.12 (0.82–1.55)
Two types of ACE	0.49	0.20	6.18	<0.05	1.64 (1.11–2.42)
Three or more types of ACE	0.61	0.20	9.59	<0.01	1.84 (1.25–2.71)
^a^ Substance misuse					
One type of ACE	0.17	0.14	1.55	0.21	1.19 (0.91–1.56)
Two types of ACE	0.34	0.16	4.63	<0.05	1.41 (1.03–1.93)
Three or more types of ACE	0.63	0.16	16.26	<0.01	1.87 (1.38–2.54)
^a^ Worse life functioning					
One type of ACE	0.29	0.18	2.51	0.11	1.33 (0.93–1.89)
Two types of ACE	0.31	0.21	2.32	0.13	1.37 (0.91–2.04)
Three or more types of ACE	1.21	0.26	21.67	<0.01	3.37 (2.02–5.61)

ACEs, adverse childhood experiences; CI, confidence interval; NA, not applicable. ^a^ Age at inclusion and sex were included as covariates in the analyses.

**Table 6:** Age at onset and scores of the Diagnostic Interview for Genetic Studies of clinical outcomes in the group with adverse childhood experience compared to groups with adverse post-childhood experience and without adverse experience.

Group According to ACEs Type(Frequency, %)	AAO (Years)	Chronicity	Psychosis	Mixed Symptoms	Suicidality	Worsening Function	Substance Misuse
Parental loss (7.8)	19.57	2.53	2.34^**^	0.84	2.40	2.61^a,b,**^	2.35
Sibling loss (5.3)	17.44	2.54	2.19^a**^	0.82	2.23^b,**^	2.56	2.30
Chronic illness (10.3)	16.29^a,b,**^	2.55	2.28^**^	0.87	2.44	2.51^b,**^	2.24
Lengthy hospitalization (10.4)	16.37^a,**^	2.58^a,*^	2.39^**^	0.79	2.40^b,**^	2.59^a,b,**^	2.39^a,**^
Permanent injury or disability (4.8)	17.69	2.59^a,b,**^	2.47^a,**^	0.85	2.42^b,**^	2.86^a,b,**^	2.30^b,*^
Physical abuse (39.4)	16.54^a,**^	2.54^a,**^	2.16^a,c**^	0.87^**^	2.60^a,b,**^	2.54^a,**^	2.39^a,**^
Injury or death threat (21.3)	16.23^a,*^	2.58^a,b,**^	2.40^a,**^	0.89^**^	2.67^a,b,**^	2.63^a,b,**^	2.46^a,**^
Leaving home unexpectedly (16.0)	17.09	2.57^a,b,**^	2.44^a,**^	0.81^*^	2.47^*^	2.53^a**^	2.33^a,**^
Serious unexpected life change (36.5)	17.16	2.56^a,**^	2.27^**^	0.84^**^	2.49^**^	2.50^a**^	2.31^a,**^

^*^p < 0.05, ^**^ *p* < 0.01. AAO, age at onset; CLES, childhood life events scale; ACEs, adverse childhood experiences; n.s, non-significant. ^a^ Multiple comparisons (Tukey) indicated that the group with adverse childhood experience had worse clinical outcomes than did those in the group without adverse childhood experience. ^b^ Multiple comparisons (Tukey) indicated that the group with adverse post-childhood experience had worse clinical outcomes than did those in the group without adverse childhood experience. ^c^ Multiple comparisons (Tukey) indicated that the group with adverse childhood experience had worse clinical outcomes than did those in the group with adverse post-childhood experience.

**Table 7:** Individual odds ratios for the presence of childhood physical abuse or post-childhood physical abuse and clinical outcomes of BD compared to the group without physical abuse (reference).

Variable (Categorical)	Coefficient	SE	Wald	*P*	OR (95% CI)
^a^ Psychotic features					
Post-childhood PA	0.23	0.27	6.65	0.38	1.26 (0.75–2.13)
Childhood PA	1.65	0.15	33.22	<0.01	5.19 (3.89–6.92)
^a^ Mixed symptoms					
Post-childhood PA	0.47	0.27	10.65	0.081	1.60 (0.94–2.71)
Childhood PA	0.49	0.12	8.10	<0.01	1.65 (1.30–2.08)
^a^ Suicide attempts					
Post-childhood PA	0.92	0.41	0.50	<0.05	2.50 (1.12–5.59)
Childhood PA	0.49	0.20	6.18	<0.01	1.97 (1.46–2.65)
^a^ Substance misuse					
Post-childhood PA	0.33	0.27	1.55	0.23	1.39 (0.82–2.38)
Childhood PA	0.51	0.12	4.63	<0.01	1.66 (1.32–2.11)

PA, physical abuse. ^a^ Age at inclusion and sex were included as covariates in the analyses.

**Table 8:** Results of multiple linear regression analysis of clinical outcomes associated with adverse childhood experiences and physical abuse.

Outcomes (Continuous Variables)	Coefficient	SE	*t*	*P*	Goodness of Fit
^a^ AAO					
Number of ACE	−0.78	0.42	−4.25	<0.01	
PA	−2.06	0.57	−3.61	<0.01	*R^2^* = 0.043
^b^ Chronicity					
Number of ACE	0.038	0.013	3.05	<0.01	
PA	0.03	0.037	0.81	0.42	*R^2^* = 0.068
^b^ Psychotic episode					
Number of ACE	0.42	0.025	16.92	<0.01	
PA	0.11	0.074	1.42	0.16	*R^2^* = 0.28
^b^ Mixed symptoms					
Number of ACE	0.017	0.021	0.79	0.43	
PA	0.13	0.062	2.07	<0.05	*R^2^* = 0.041
^b^ Suicidality					
Number of ACE	0.084	0.032	2.58	<0.01	
PA	0.23	0.097	2.42	<0.05	*R^2^* = 0.072
^b^ Substance misuse					
Number of ACE	0.069	0.026	2.60	<0.01	
PA	0.18	0.080	2.26	<0.05	*R^2^* = 0.044
^b^ Worsening function					
Number of ACE	0.10	0.020	5.06	<0.01	
PA	0.058	0.059	0.97	0.33	*R^2^* = 0.075

AAO, age at onset; ACEs, adverse childhood experiences; PA, physical abuse. ^a^ Sex was included as a covariate in the analyses. ^b^ Age at inclusion, sex, and AAO were included as covariates in the analyses.
